# Clear Cell Chondrosarcoma in Association With Niemann-Pick Disease

**DOI:** 10.1080/13577140500090030

**Published:** 2005

**Authors:** K. N. Srikanth, A. Kulkarni, A. M. Davies, V. P. Sumathi, R. J. Grimer

**Affiliations:** 1 Department of Oncology The Royal Orthopaedic Hospital Birmingham United Kingdom; 2 MBBS, FRCS, FRCS (Orth) The Royal Orthopaedic Hospital Bone Tumour Service Bristol Road South Birmingham B31 2AP United Kingdom

## Abstract

*Purpose:* The purpose of this case report is to bring to light this unusual combination of two rare diseases, namely Neimann-Pick disease Type B and clear cell chondrosarcoma occurring in the same patient. This has not previously been reported in the world literature.

*Subject:* Niemann-Pick disease (NPD) is a rare autosomal recessive inborn error of metabolism. Type B NPD is even rarer. It is a lysosomal storage disorder affecting children and adolescents often causing death in early childhood, although in milder form patients may survive up to adulthood, like our patient. Clear cell chondrosarcoma is a very rare type of chondrosarcoma affecting the epiphyseo-metaphyseal region of long bones. We present a patient suffering from a milder form of Neimann Pick disease who developed a clear cell chondrosarcoma. We investigated to find if there was likely to be any relationship between these two events.

*Results:* NPD type B is caused by a three-base deletion in chromosome 11. Chondrosarcoma and multiple exostoses occur due to loss of tumour suppressor gene EXT 2 from centromeric region on chromosome 11, though it is difficult to establish the link between the two, as the two together have not yet been reported in the literature. NPD may present diagnostic difficulties when it occurs with chondrosarcoma.

*Discussion:* We conclude that the two diseases have not been reported together in the world literature and there is some evidence to show that chromosome 11 is central to both diseases. More research is needed to see if one leads to the other.


*Sarcoma*, March/June 2005; 9(1/2): 33–36

Taylor & Francis

Taylor & Francis Group

**CASE REPORT**

### Clear cell chondrosarcoma in association with Niemann-Pick disease

K. N. SRIKANTH, A. KULKARNI, A. M. DAVIES, V. P. SUMATHI, & R. J. GRIMER

*Department of Oncology, The Royal Orthopaedic Hospital, Birmingham, UK*

**Abstract**
*Purpose*: The purpose of this case report is to bring to light this unusual combination of two rare diseases, namely Neimann-Pick disease Type B and clear cell chondrosarcoma occurring in the same patient. This has not previously been reported in the world literature.
*Subject*: Niemann-Pick disease (NPD) is a rare autosomal recessive inborn error of metabolism. Type B NPD is even rarer. It is a lysosomal storage disorder affecting children and adolescents often causing death in early childhood, although in milder form patients may survive up to adulthood, like our patient. Clear cell chondrosarcoma is a very rare type of chondrosarcoma affecting the epiphyseo-metaphyseal region of long bones. We present a patient suffering from a milder form of Neimann Pick disease who developed a clear cell chondrosarcoma. We investigated to find if there was likely to be any relationship between these two events.
*Results*: NPD type B is caused by a three-base deletion in chromosome 11. Chondrosarcoma and multiple exostoses occur due to loss of tumour suppressor gene EXT 2 from centromeric region on chromosome 11, though it is difficult to establish the link between the two, as the two together have not yet been reported in the literature. NPD may present diagnostic difficulties when it occurs with chondrosarcoma.
*Discussion*: We conclude that the two diseases have not been reported together in the world literature and there is some evidence to show that chromosome 11 is central to both diseases. More research is needed to see if one leads to the other.

### Introduction

Niemann-Pick disease (NPD) is a rare autosomal recessive inborn error of metabolism, a lysosomal storage disorder characterized by deficiency of acid sphingomyelinase. Acid sphingomyelinase is an enzyme metabolising cell membrane lipid sphingomyelin. This leads to sphingomyelin deposition in the lysosomes of cells in brain, reticuloendothelial and lung tissue [1]. Chondrosarcoma is a rare slow-growing malignant tumour of bone producing cartilage matrix, common in the the fifth and sixth decades [2]. These two diseases have not been reported together as patients with NPD usually die early and chondrosarcoma is common after the fifth decade.

### Case report

A 50-year-old right-handed maintenance engineer presented with a 4-week history of left elbow pain. It was initially diagnosed as tennis elbow but no X-rays were taken. He later sustained a pathological fracture of his left olecranon while opening a door (Figure 1). This fracture was internally fixed by the referring hospital but a sample taken at the time of surgery revealed a chondrosarcoma and hence he was sent to the oncology service at our hospital.

He had a history of NPD as a child and had splenomegaly and interstitial lung disease but otherwise had no signs of manifestation of the disease.

Correspondence: Mr Robert J. Grimer, MBBS, FRCS, FRCS (Orth), Bone Tumour Service, Royal Orthopaedic Hospital, Bristol Road South, Birmingham B31 2AP, UK. Tel: 44 121 6854000. Fax: 44 121 6854146. E-mail:rob.grimer@roh.nhs.uk

ISSN 1357–714X print/ISSN 1369–1643 online © 2005 Taylor & Francis Group Ltd
DOI: 10.1080/13577140500090030

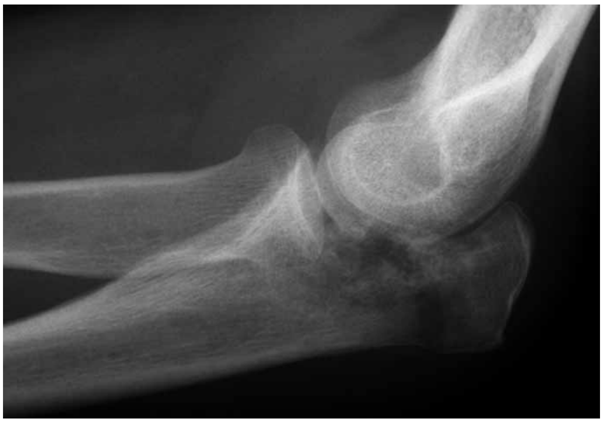

Figure 1. Lateral Radiograph elbow. This shows a pathological fracture of the olecranon. Features are that of an aggressive lesion but are otherwise non-specific.

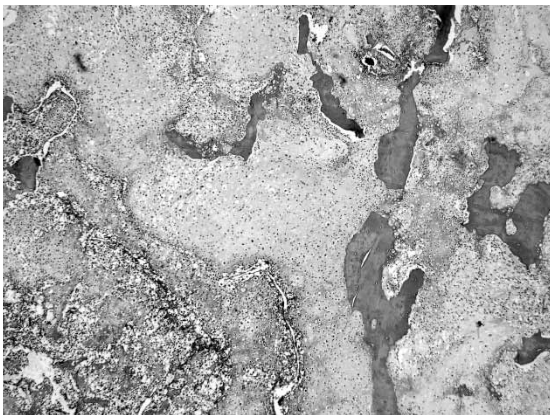

Figure 2. Low power photomicrograph. This shows areas of grade II conventional chondrosarcoma (right half of the field) invading host bony trabeculae and showing clear cell change (left half of the field).

Examination of his left elbow showed a range of 30–90° of flexion with full supination and pronation. Neurovascular status of the left upper limb was normal. Magnetic resonance imaging of his left elbow showed an effusion in the joint and not much else due to the implant. Computer tomography and bone scintiagraphy showed no evidence of metastases.

The patient opted for an above elbow amputation because of the high risk of local recurrence with limb salvage surgery especially in view of the intra-articular disease and previous surgery. Histology of the amputated limb showed a grade II conventional chondrosarcoma mixed with foci of clear cell chondrosarcoma of the left proximal ulna with wide amputation margins (Figure 2). The patient has subsequently developed multiple lung metastases.

### Results

We did a literature search to understand the nature of the two diseases, probed if there were any genetic link between the two and if one can influence the presentation, progression and prognosis of the other. NPD type B is caused by delta 608 mutation, a three-base deletion in chromosome 11 that causes the removal of an arginine residue from position 608 of the ASM polypeptide [3]. Chondrosarcoma and multiple exostoses occur due to loss of tumour suppressor gene EXT 2 from centromeric region on chromosome 11 by base deletion as one of the mechanism [4], though it is difficult to establish the link between the two with available research in molecular genetics. Though we could not prove that one can influence the progression and prognosis of the other, certainly NPD produces interstitial lung disease which could present diagnostic difficulties in ruling out pulmonary metastases by imaging, as happened in our patient where initially diagnosed interstitial lung disease in the follow-up CT proved

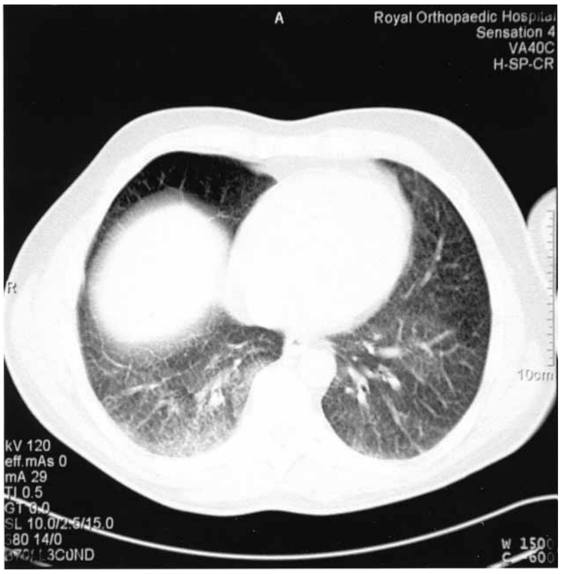

Figure 3. Computed tomography of the chest. This shows lung window images of lung bases showing reticulo-nodular pattern. These ill-defined changes are suggestive of interstitial lung disease though miliary metastases are difficult to rule out.

to be lung secondaries from chondrosarcoma (Figures 3 and 4).

### Discussion

NPD is an inborn error of metabolism, leading to deposition of lipid in the lysosomes of cells [1,5]. Histologically large pale foamy cells are seen in the reticuloendothelial system, which stain positively with lipid stains. The gene for acid sphingomyelinase is carried on chromosome 11, mutations of which cause decreased production of the active enzyme [3,6].

There are three types of NPD. Type A disease has less than 5% of active enzyme, and is a rapidly progressive neurodegenerative disease of infancy

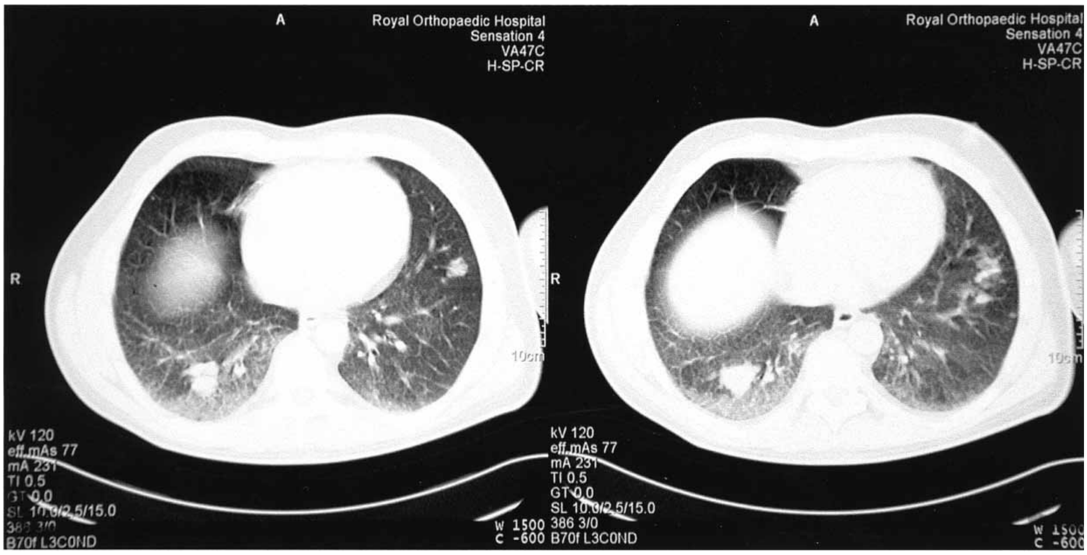

Figure 4. Computed tomography of the chest. This shows lung window images of the bases showing irregular nodular lesions measuring up to 2 cm in diameter, suggestive of pulmonary metastases. Chondrosarcoma usually produces macro nodular metastases.

manifested by failure to thrive, severe psychomotor retardation, feeding problems, progressive spasticity, blindness with cherry red spot, hepato-splenomegaly, jaundice of infancy with progressive liver failure and death. Most patients die by the age of 2–3 years.

Type C is also a severe form and due to defective cholesterol transport, trapping sphingomyelin and cholesterol inside cells with central nervous system involvement leading to seizures and death by 5–15 years of age.

Type B, as seen in this case, is a milder form with 5–10% of acid sphingomyelinase activity and patients live up to late childhood and adulthood, like our patient. It is characterised by reticuloendothelial system sphingomyelin deposition leading to hepato-splenomegaly and pulmonary involvement with an absence of neurological manifestation and survival into adulthood [6,7].

Diagnosis of NPD is by enzyme assay of acid sphingomyelinase from peripheral blood WBCs or from cultured skin fibroblasts. Prenatal diagnosis by amniocentesis is possible. At present most of the available treatments are preventive [8], supportive or experimental. These include enzyme replacement therapy, gene therapy, and bone marrow transplant.

Chondrosarcoma is a rare malignant bone tumour producing cartilage matrix common in the fifth and sixth decade with a male to female ratio of 3:2. Our patient had a clear cell chondrosarcoma with associated classical grade II chondrosarcoma. Clear cell chondrosarcoma is also called ‘malignant chondroblastoma’. It represents about 2% of malignant cartilaginous bone tumours with a male to female ratio of 3:2. It commonly affects the proximal femur or tibia and only rarely the ulna. Histologically it shows sheet-like non-lobular arrangement of cartilaginous cells with tumour cells showing abundant clear cytoplasm and distinct cell borders due to paucity of organelles and a cytosol with low protein content in these cells. The presence of a spectrum of immature chondroblasts to mature chondrocytes is characteristic of clear cell chondrosarcoma.

Radiologically it shows as an ephiphyseal osteolytic and expansile lesion. More than 50% of these tumours do not show calcification. As soft tissue invasion is rare this tumour has a good prognosis. A combination of clear cell and classical chondrosarcoma is not uncommon, whilst dedifferentiated clear cell chondrosarcoma has also been reported [10].

The treatment of chondrosarcoma is usually surgical with wide local excision. Radiotherapy and chemotherapy are not effective. If the tumour is incompletely excised it has a high incidence of local recurrence and subsequent metastasis [11,12].

It was difficult to prove that one leads to the other, though more research in molecular genetics probing this problem may well establish a link in the future. Although chondrosarcoma is associated with altered carbohydrate metabolism [12], we could not prove that one can influence the progression and prognosis of the other; however, NPD does produce interstitial lung disease, which could present diagnostic difficulties in ruling out pulmonary metastases. With the presently available evidence, the two diseases arising together appears to be a chance occurrence.

### References

1. Brady RO. The sphingolipidoses. New Engl J Med 1966; 275:312–318.
2. Giudici MA, Richard P, Moser P, Marks J, Kransdorf. Cartilaginous bone tumors, Radiol Clin N Am 1993;31(2): 237–259.

3. Levran O, Desnick RJ, Schuchman EH. Niemann-Pick type B disease: Identification of a single codon deletion in the acid sphingomyelinase gene and genotype/phenotype correlations in type A and B patients. J Clin Invest 1991;88: 806–810.
4. Raskind WH, Conrad EU, Chansky H, Matsushita M. Heterozygosity in chondrosarcomas for markers linked to hereditary multiple exostoses loci on chromosomes 8 and 11. Am J Hum Genet 1995;56:1132–1139.
5. Brady RO, Kanfer JN, Mock MB, Fredrickson DS. The metabolism of sphingomyelin II. Evidence of an enzymatic deficiency in Niemann-Pick disease. Proc Natl Acad Sci USA 1966;55:366–369.
6. Levran O, Desnick RJ, Schuchman EH. Niemann-Pick disease: a frequent missense mutation in the acid sphingomyelinase gene of Ashkenazi Jewish type A and B patients. Proc Natl Acad Sci USA 1991;88:3748–3752.
7. Lowden JA, Laramee MA, Wentworth P. The sub acute form of Niemann-Pick disease. Arch Neurol 1967;17:230–237.
8. Wenger DA, Wharton C, Sattler M, Clark C. Niemann-Pick disease: Prenatal diagnosis and studies of sphingomyelinase activities. Am J Med Genet 1978;2:345–356.
9. Bullough PG. Orthopaedic Pathology, 3rd edn. London: Times Mirror International Publishers Limited; 1997. pp 313–377.
10. Kalil RK, Inwards CY, Unni KK, Bertoni F, Bacchni P, Wenger DE, Sim FH. De differentiated clear cell chondrosarcoma. Am J Surg Pathol 2000;24:1079–1086.
11. Mirra JM, Picci P, Gold RH, Joseph M. Mirra's bone tumours, clinical, radiologic, and pathologic correlations, 1st edn. New York: Lea and Febiger, 1989. pp 535–546.
12. Huvos A. Bone tumours, diagnosis, treatment and prognosis, 2nd edn. New York: W.B. Saunder, 1991. pp 313–373.

